# Prevalence and Associated Factors of Suicidal Behavior Among Patients and Residents in Northwest Ethiopia

**DOI:** 10.3389/fpsyt.2021.560886

**Published:** 2021-09-27

**Authors:** Habte Belete, Eyaya Misgan, Tilahun Belete

**Affiliations:** ^1^Department of Psychiatry, College of Medicine and Health Sciences, Bahir Dar University, Bahir Dar, Ethiopia; ^2^Department of Gynecology and Obstetrics, College of Medicine and Health Sciences, Bahir Dar University, Bahir Dar, Ethiopia

**Keywords:** suicidal behavior, medical illness, primary health care, low-income, Ethiopia, mental health

## Abstract

There are a million suicide deaths in the world annually, and 75% of these occur in low- and middle-income countries (LMICs). However, there are limited resources to prevent suicidal deaths in those regions. The aim was to assess the prevalence of suicidal behavior and associated factors among patients visiting for medical care at a health center and residents in the community. A comparative study was employed by interviewing 2,625 residents in the community and 1,363 patients at the health center about suicidal behavior in northwest Ethiopia, from March 2017 to February 2018. Logistic analysis was employed with adjusted odds ratios and 95% confidence interval (CI) and with *p*-value < 0.05. The total prevalence of suicidal behavior (ideation, plan, and attempt) was found to be 5.6% (with 95% CI range 5–6%). It was found to be 4.4% with 95% CI range 4–5% in residents and 7.9% with 95% CI range 6–9% in patients. Female sex, depressive symptoms, physical/verbal abuse, and feeling stigmatized were identified as a risk factors for suicidal behavior, whereas a healthy lifestyle, such as eating regular meals of fruits and vegetables, doing physical exercise regularly, and having public health insurance were identified as protective risk factors for suicidal behavior even after adjusting for being a patient or not. The proportion of suicidal behavior was double in patients compared with residents. Suicidal behavior should be assessed in patients who visit for medical help and integration of mental health service within the primary health care system is recommended, especially in low-income countries.

## Introduction

Every year, there are a million suicide deaths in the world, and of this, two-thirds occur in low- and middle-income countries (LMICs), including Ethiopia (1), where resources to prevent suicidal deaths are limited (2). However, only 6% of the data on suicide emerges from those regions (3), and there is a lack of national systematic reporting for case-specific mortality.

Suicide is the second leading cause of death, especially in adulthood, 15- to 29-year-olds (4), and for each suicidal death in adulthood, there may have been more than 20 others attempting suicide (2). Recently, due to its rigorous effect across the world, the World Health Organization (WHO) made a plan to reduce the rate of suicide by 10% in 2020 (5). In developing regions, particularly in sub-Saharan areas, due to stigma, traditional belief, and cultural rituals, suicide may be an unknown cause of deaths in culturally diverse rural communities (6–8) and evidenced by people who committed suicide considered sinners in society (6) in Ethiopia.

Even though the World Health Organization has a plan of indicators to measure suicide death incidence (2), it still produces few reports on the magnitude of suicide in rural residents and patients in resource-limited countries with high a magnitude of morbidities. Moreover, the link between medical or somatic disorders and suicidal behavior remains unclear. Though a majority of patients could visit a health care facility, only 24% of them had received a mental health diagnosis before committing suicide (9). People with diagnoses of cancer are at increased risk of suicide, especially in the 3 months following initial diagnosis (10). Cancers at an advanced age at diagnosis were associated with an increased risk of suicide within 1 year of diagnosis, which encompasses up to 40% of total suicides (11). Primary care is an important setting for suicide prevention. A study on U.S. veterans describes that thoughts of taking one's life were endorsed by 24% of participants, and suicide attempts were reported by 2% of them (12). The 1-month prevalence of suicidality at an outpatient clinic in China was 2.3% (13); 2–3% in an African review showing also that socioeconomic characteristics of patients correlates with suicidal behavior, and lifetime suicide attempts were reported by 3.2% (*n* = 332) of the study population (3). Of these, 63% (*n* = 208) were women (14, 15). The overall 12-month prevalence of non-fatal suicidal behavior, consisting of suicidal ideation, plan, and attempt, was 7.9% [95% confidence interval (CI) = 6.8–8.9%] in Ethiopia (16).

Suicidal behavior prevalence seems different in residents in the community and patients who visit a health center. A comparative study reports on the prevalence of non-fatal suicidal behavior, consisting of suicidal ideation, plan, and attempt, as significantly higher in patients (10.3%) compared with residents (6.3%). However the 12-month prevalence of suicide attempts was 4.4% (95% CI = 3.6–5.3%), non-significantly higher among patients (5.4%) compared with residents (3.8%) in rural Ethiopia (16). Risk factors associated with suicidal behavior include being female, a younger age, a current mental disorder, and having lower educational and economic status as described in low-income countries (17).

Our hypothesis was that the prevalence of suicidal behavior is higher in patients than residents. Therefore, to answer our hypothesis, the aim of this study was to assess suicidal behavior prevalence and identify associated factors among patients at the primary health care setting and residents in the community.

## Methods

### Study Setting

The study was conducted at Merawi Health Center and in the community that is a semi-urban and rural area in northwest Ethiopia in 2018. This health center delivers a range of non-referral primary services, including antenatal and postnatal care, chronic diseases, HIV/AIDS, hypertension, and diabetes cases for more than 81,000 people. However, mental health care is not delivered at the health center nor with liaison psychiatry in the community, but it is available in informal settings such as with holy water (directed by spiritual healers) and traditional medicines (settings under the supervision of herbalists or traditional healers) in the community.

### Study Population and Group Samples

The study population are adult permanent residents in the community and selected patients who visited for medical care at Merawi Health Center during the study period.

### Inclusion and Exclusion Criteria

Inclusion criteria were being an adult age 18 years and above belonging to the study population, a random selection of residents who permanently reside in the community (for at least 6 months) and of adult patients who visited for medical care at Merawi Health Center. Participants who received diagnoses of depression before the survey and participants who could not communicate well for data collectors were excluded.

The number of participants was calculated by EPI-INFO version 7 with the assumption of a double population formula at margin of error of 5%, statistical power of 90%, and 95% CI. The proportion of suicidal behavior in residents was 6.9%, in health facility visitors 10% (16), and the ratio of patients to residents was 1:2 to improve the sample representativeness of the population. By adding a 10% non-response rate, the final sample size expected was 4,289. This survey includes a large number of participants, both community residents and patients at the primary health care level.

### Method of Selection of Participants and Setting Interviews in the Community and at the Medical Health Center

A systematic random sampling method was used to select residents at the household level. The first household was selected by a lottery method from the given interval, and then one randomly selected member of the family was taken if there was more than one eligible member in one household. A well-trained nurse and data collectors visited at a convenient time to get participants for the interview, and if they were not available at their home, appointments were given two times before they reached the level of non-response.

The patients were also selected randomly at first and then within a fixed skip interval at the outpatient department at the Merawi primary health center. They were interviewed by trained nurses in a separate room for privacy.

### Instruments

Suicidal behavior (ideation, plan, and attempt) has been assessed with the Suicide Behaviors Questionnaire-Revised (SBQ-R), which is suitable for such study as described in detail previously (18). The SBQ-R contains four items on suicidal behaviors, and the total score of the SBQ-R ranges from 3 to 18. For our study a score ≥7 was considered to be suicidal behavior with a sensitivity of 0.93 and specificity of 0.95 that indicate the risk of suicide in the general adult population. The SBQ-R is also approved as a useful scale for suicide risk assessment in clinical and non-clinical samples (19).

Depression was assessed with a score ≥8 on the Hospital Anxiety and Depression Scale (HADS). Hospital Anxiety and Depression Scale is a 14-item tool used to screen for symptoms of anxiety and depression, and it is separated into two 7-item subscales. In this study, we report on depressive symptoms. Hospital Anxiety and Depression Scale has good validity and reliability for screening depression and was validated in Ethiopia, and it also has an internal consistency 0.76 for depression subscales (20). Current substance use was assessed based on the participant's report of use of either alcohol, smoking, cannabis, or khat at least once in the past 3 months (21). The level of support was assessed by using three items of the Oslo-3 social support scale (22), which helps to assess the number of close confidantes, perceived level of concern from others, and perceived ease of getting help from neighbors. Participants were asked to rate their feeling about whether they are feeling stigmatized or not by the community. The presence of any physical/verbal abuse in the person's lifetime was assessed based on the abuse assessment screen assumptions for verbal or physical abuse by asking participants if they have ever been emotionally or physically abused by someone, including experiencing being pushed, hit, slapped, physically hurt or kicked, punched, threatened with abuse, bruises, cuts, and/or continuing pain, being beaten up, severe contusions, burns, broken bones, verbal threats, or aggressive shouting.

A healthy lifestyle was assessed based on participants' responses regarding whether or not they were doing a regular physical exercise (two to three times) in the week and having fruits/vegetables in their regular meal (two to three times) per week to improve wellness as it becomes a common practice to include available fruits and vegetables into the regular meal in Ethiopia. Participants' relative wealth was assessed by simply asking the participants how they perceived their wealth compared with other people in the neighborhood (23). To be statistically more powerful, we kept two levels of wealth: lower own income vs. same or above. Educational status was classified into two categories, educated vs. uneducated, by considering participants' elementary level of education and above as educated. Current marital status was grouped into two categories, married vs. unmarried, by considering single, separated, divorced, and widowed as currently unmarried. We asked participants whether or not they use health insurance for their family. Currently, the cost of health care is increasing in Ethiopia and seems unaffordable with out-of-pocket expenses to improve the health-seeking behavior of the patients. The Ethiopian government has launched a public health insurance service. However, there still is a substantial proportion of people who do not use public health insurance, and this may affect their access to mental or physical health care.

### Analysis

The collected data was checked for completeness and consistency manually and then coded and entered in the Epi-Data 3.1 software, and finally exported to Statistical Package for the Social Sciences (SPSS) version 20 software for analysis. Outliers were checked before the regression analysis by using a boxplot during the descriptive analysis, then a chi-square test, and bivariate logistic regression analysis was performed to examine the unadjusted relationship between the predictors and dependent variables. For independent variables, which are associated with suicidal behavior at *p*-value < 0.05 during bivariate logistic regression analyses, multivariable logistic regression analysis was done by using the backward stepwise method (with three steps in our case), and *p*-value < 0.05 used to declare a statistically significant determinant of suicidal behavior. The Hosmer and Lemeshow test was used to check the model fitness, and its *p*-value was >0.05 (*p* = 0.602), and the adjusted odds ratio with a 95% CI was used to indicate the strength of association.

## Results

Among the total participants contacted for in the study, 3,988 participants (93%) were included in for data analysis in the two groups that we call “patients” and “residents”: 1,363 patients visiting the health center and 2,625 residents from the community. Risk factors of anxiety and depression in this sample as well as comparison of characteristics between the two groups of patients and residents have been published previously and are not reported here again (24).

### Socio-Demographic Characteristics of the Participants

Women accounted for 50.3%, and 73.1% of the participants were urban dwellers. The mean age of participants was 35.37 ± 13.258 years (range from 18 to 98 years). Comparing patients to resident groups, patients were more often female, with advanced age, more often from a rural area, less educated, more often jobless, more often living with the family, with a bigger family size, and less often with low wealth than residents (Table 1).

**Table 1:** Bivariate comparison of participants' sociodemographic characteristics in relation to having YES or NO suicidal behaviors.

**Characteristics**		**Whole sample**	**Suicidal behavior**	***p*-value**	**Chi-square test**
		**(*N* = 3,988)**	**(*N* = 224)** **Yes % (*N*)**		**(*X*^**2**^)**
Sex	Female	50.3% (*N* = 2,004)	57.6% (*N* = 129)	0.024	5.11
Age groups	≥45 years	24% (*N* = 958)	25.4% (*N* = 57)	0.608	0.26
Residency	Urban	73.1 (*N* = 2,916)	72.8% (*N* = 163)	0.903	0.015
Marital status	Unmarried^*^	40.1% (*N* = 1,600)	37.5% (*N* = 84)	0.410	0.68
Wealth	Lower	25.6% (*N* = 1,021)	31.3% (*N* = 70)	0.046	3.98
Education	Uneducated	24.9% (*N* = 994)	22.8% (*N* = 51)	0.442	0.59
Family size	3 and above	83.1% (*N* = 3,316)	79.5% (*N* = 178)	0.129	2.30
Living condition	Alone	6.1% (*N* = 243)	4.5% (*N* = 10)	0.294	1.10
Job status	Jobless	54% (*N* = 2,153)	52.2% (*N* = 117)	0.588	0.29

* *divorced, widowed, separated, and single*.

### Psychosocial Factors of the Participants

More than a quarter of all the participants and 24.4% of patients had no regular meal of fruits and vegetables. About 74.8% of all participants and 80.3% of patients did not participate in regular exercise. Low social support accounted for 15.0% in patients and 12.5% in residents. We noticed more health insurance, fewer reports of feeling stigmatized, with less history of physical or verbal abuse, less physical exercise, and less alcohol and drug use in the patient group compared with the resident group. The main differences in drug use between residents and patients was khat chewing reported by 133 residents and only 26 patients; cannabis was used by 13 residents and five patients (Table 2).

**Table 2:** Bivariate comparison of participants psychosocial and medical characteristics in relation to having YES or NO suicidal behavior.

**Characteristics**		**Whole sample**	**Suicidal behavior**	***p*-value**	**Chi-square test**
		**(*N* = 3,988)**	**(*N* = 224)** **Yes % (*N*)**		**(*X*^**2**^)**
Regular exercises	No	74.8% (*N* = 2,982)	86.6% (*N* = 194)	<0.0001	17.62
Fruits and vegetables	No	25.3% (*N* = 1,008)	44.6.0% (*N* = 100)	<0.0001	47.13
Social support	Low	14.2% (*N* = 566)	16.1% (*N* = 36)	0.530	1.27
Health insurance	No	45.8% (*N* = 1,826)	63.4% (*N* = 142)	<0.0001	29.64
Feeling stigmatized	Yes	9.8% (*N* = 390)	28.1% (*N* = 63)	<0.0001	90.53
Abuse	Yes	49.1% (*N* = 1,960)	57.1% (*N* = 128)	0.014	6.07
Alcohol use	Yes	26.8% (*N* = 1,069)	31.3% (*N* = 70)	0.122	2.39
Smoking	Yes	4.5% (*N* = 181)	3.1% (*N* = 7)	0.295	1.10
Khat chewing	Yes	4% (*N* = 159)	2.7% (*N* = 6)	0.314	1.02
Any substance^*^	Yes	31.3% (*N* = 1,247)	34.8% (*N* = 78)	0.238	1.39
Depression	Yes	6.7% (*N* = 269)	16.1% (*N* = 36)	<0.0001	32.82
Being from the patients' group	Yes	34.2% (*N* = 1,363)	48.2% (*N* = 108)	<0.0001	20.79

* *khat, alcohol, smoking, and cannabis*.

### Comparison of Suicidal Behavior Between Residents and Patients

The total prevalence of suicidal behavior was 5.6% (with 95% CI range 5–6%). It was found to be 4.4% (with 95% CI range 4–5%) in residents and 7.9% (with 95% CI range 6–9%) in patients. The proportion of suicidal behavior was more than double in participants with depressive symptoms (13.4% with 95% CI range from 9.0 to 17.0%) than participants without depressive symptoms (5.1% with 95% CI range from 4.0 to 6.0%) (Table 3).

**Table 3:** Multivariable logistic regression of risk factors associated with suicidal behavior in the whole sample (*N* = 3,988).

**Variables**		**Suicidal behavior**		**AOR**	**p-value**
		**Yes**	**No**		
		***N* (%)**	***N* (%)**		
Sex	Female	129 (57.6)	1,875 (49.8)	1.42 (1.07, 1.89)	
	Male	95 (42.4)	1,889 (50.2)	Ref	0.016
Feeling stigmatized	Yes	63 (28.1)	327 (8.7)	3.70 (2.65, 5.15)	
	No	161 (71.9)	3,437 (91.3)	Ref	<0.0001
Abuse	Yes	128 (57.1)	1,832 (48.7)	1.34 (1.01, 1.80)	
	No	96 (42.9)	1,932 (51.3)	Ref	0.047
Depressive symptoms	Yes	36 (16.1)	233 (6.2)	1.67 (1.11, 2.52)	
	No	188 (83.9)	3,531 (93.8)	Ref	0.032
Fruits and vegetable in meal	Yes	124 (55.4)	2,856 (75.9)	0.42 (0.31, 0.56)	
	No	100 (44.6)	908 (24.1)	Ref	<0.0001
Regular exercise	Yes	30 (13.4)	976 (25.9)	0.38 (0.25, 0.58)	
	No	194 (86.6)	2,788 (74.1)	Ref	<0.0001
Use of health insurance	Yes	82 (36.6)	2,080 (55.3)	0.45 (0.34, 0.60)	
	No	142 (63.4)	1,684 (44.7)	Ref	<0.0001
Wealth	Lower	70 (31.3)	951 (25.3)	1.26 (0.93, 1.71)	
	≥Same	154 (68.8)	2,813 (74.7)	Ref	0.141
Sample	Patients	108 (48.2)	1,255 (33.3)	2.16 (1.62, 2.89)	
	Residents	116 (51.8)	2,509 (66.7)	Ref	<0.0001

*AOR, Adjusted odds ratio; Ref, Reference groups*.

### Number of Suicidal Behavior and Depressive Symptoms in Different Medical Disorders in the Patient Group

The type of illness was recorded only in the patient group visiting the health center with number of patient illness (respectively, number with suicidal behavior or with depressive symptoms), 235 patients presenting with hypertension (*N* = 20 with suicidal behavior, *N* = 27 with depressive symptoms), 184 with diabetes (*N* = 16, *N* = 19), 340 with HIV/AIDS (*N* = 26, *N* = 47), 136 surgical illness (*N* = 7, *N* = 9), 413 other medical illnesses (including epilepsy, respiratory, gastrointestinal symptoms, etc.) (*N* = 35, *N* = 28), and 56 other mental illnesses, including developmental and behavioral disorders (*N* = 4, *N* = 7) (descriptive results only).

### Statistical Analysis for Risk Factors Associated With Suicidal Behavior

The 224 participants with suicidal behavior, compared in bivariate analysis, to other participants of the study, were more often women, 57.6% (*p* = 0.024); more often feeling stigmatized, 28.1% (*P* < 0.0001); more often not having fruits and vegetables in their regular meal, 55.4.0% (*p* < 0.0001); with no health insurance, 63.4% (*p* < 0.0001); with a history of abuse, 57.1% (*p* = 0.014); more often did not do regular exercises (*p* < 0.0001); more often with low perceived wealth, 31.3% (*p* = 0.047); more often depressive symptoms, 16.1% (*p* < 0.0001); and more often from the patient group, 48.2% (*p* < 0.0001). However, the following factors did not reach statistical significance at a *p*-value < 0.05 and were not included as risk factors in the multivariate logistic regression model: urban location, low social support, having a big family size, being jobless, advanced age of 45 and above, being unmarried, being uneducated, and substance use (alcohol, smoking, and khat chewing and cannabis).

After testing all variables in relation to suicidal behavior at bivariate analyses, female sex, abuse (physical/verbal), perceived wealth, having depressive symptoms, and feeling stigmatized were identified as risk factors, whereas having health insurance for the family, doing regular exercise, and eating fruits and vegetables at regular meals were protective factors. Then, these factors were considered for further analysis because they were candidates for multivariable logistic regression at a significance level of *p* < 0.05.

Then, multivariable logistic regression was done to see the adjusted odds ratio (after controlling for confounding factors of being from the resident or the patient group) at a significance level of *p* < 0.05, and all the above variables included in the model reach statistical significance (at *p*-value < 0.05) for explaining suicidal behavior except the relative perceived wealth factor, which was not significant (Table 3).

## Discussion

Suicidal prevention becomes an emerging priority for health care prevention. Even though a lot of suicide deaths have been reported recently or currently under care in clinical settings, suicide prevention has not been seen as a core priority in the health care system (25). Local evidence is important to support suicidal prevention in the community and to integrate mental health services into the primary health care level in resource-limited settings. The prevalence of suicidal behavior is found to be double among patients compared with residents. It has a similar message to prior studies done in LMICs, including Ethiopia, Uganda, South Africa, India, and Nepal in which suicidal behavior was 10.3% in health care attendees and ranges from 3.5 to 11.1% in the residents in the community (17).

This study reveals protective and risk factors of suicidal behavior among residents and patients analyzed in a multivariable logistic regression. The factors associated with suicidal behavior include female sex, depressive symptoms, being from the patient group, abuse (physical/verbal), and feeling stigmatized. Having a regular meal of fruits and vegetables, doing physical exercises regularly, and use of health insurance for one's family were identified as protective factors for suicidal behavior.

However, urban residents, low social support, having a big family size, joblessness, advanced age of 45 and above, unmarried, uneducated, and substance use (alcohol, smoking, and khat chewing and cannabis) did not reach statistical significance at *p* < 0.05. The main reason might be that the number of respondents was very small for some demographic factor, such as uneducated, low social support, and advanced age 45 and above, whereas we had a very large number of respondents for bigger family size, urban residents, and joblessness unlike similar studies in the region (26). Cultural aspects of transparency for personal, religious perspectives, and culturally sensitive factors might be undermining the reporting rate of these factors, which is evidenced by low reporting of substance use in this study, specifically smoking and khat chewing. In this area, smoking and khat chewing are highly discouraged within the community (27).

Women are more at risk of suicidal behavior than men, which is in line with previous studies in LMICs, including Ethiopia (13, 16, 17, 28–30). Suicidal behavior in women within most reproductive stages (age 25–34 years) who came for postnatal routine visits at the obstetric care center in the same region in Northwest Ethiopia even show a higher prevalence of 14% of suicidal behavior during the postpartum period (31). Even though the association between education status and suicidal behavior is not significant in this study, other studies on suicide observed that educational disparity (socioeconomical disparities) in suicide is greater in men than in women (32). Educational inequality is a risk of suicidal behavior at the lower level, but no difference was observed in secondary and above education levels.

Depression is one of the main factors linked to suicidal behavior in this study. This finding is supported by previous studies (12, 13, 16, 29, 30, 33). Medical illness as a biological or related psychosocial risk factor for suicidal behavior is reported in earlier studies (17, 34) and as we observed in the patient group in this study, but this was not statistically tested here. It would be interesting to do further studies on this relation to help prevent suicidal behaviors. We found abuse (physical/verbal) to be a risk factor for suicidal behavior, and this is supported by a study done in Ethiopia that physical hurt is a risk of suicidal behavior (35, 36). This is in line with a previous study showing that sexual abuse is also a main risk factor for suicidal behavior in women in the postnatal period in the same study region of Ethiopia (31) and for depression (16, 37). Perceived medical-related stigma is a risk for suicidal behavior, and all physical health conditions increased suicide risk (34). Perceived medical-related stigma in HIV or tuberculosis patients also had a risk for suicidal behavior, which is supported by previous studies in Ethiopia (29, 38). Feeling stigmatized may make one's life wired and boring, which may increase suicidal behavior, and this could be the possible reason for the association.

A healthy eating lifestyle, such as having fruits and vegetables in the regular meal decreases the risk of suicidal behavior. Positive eating habits can be viewed as a form of self-care as it requires knowing the importance of the body with care and respect by improving mental wellness (39). This can be explained from the point of view that fruit intake helps to promote mental wellness and is associated with prevention of suicidal behavior (40, 41). Moreover, diets low in fruits and vegetables resulted in poor mental health status, such as increased depression (42). Doing regular exercise decreases the risk of suicidal behavior in this study, which is in line with an intervention study done in Australia in which mountain hiking exercise had lowered levels of hopelessness and depression, which resulted in a significant reduction in suicidal ideation over time (43). Physical exercise is a promising protective factor for suicidal behavior and related mental health problems (44–46). These studies indicate that people who are physically active tend to be at lower risk for suicidal behavior than those who are inactive; however, such results may not be universal (47). Use of health insurance for one's family was also found to decrease risk for suicidal behavior, which is supported by a previous study showing that health insurance coverage for the family is an important asset to feel secure and to decrease risk of suicidal behavior (48).

The strength of this study is its generalizability, which includes a large sample from both residents and patients and from urban and rural areas big enough to assess several associated factors in a statistically powerful logistic regression model for risk of suicidal behavior. The study was done in a low-income country where the suicide rate is high and, therefore, where such studies are needed; the factors studied are relevant to the country and are not only negative risk factors, but also protective risk factors for suicidal behavior. However, we have a number of limitations, such as the study was done in a single area of Ethiopia and only one time, which limits generalization for the whole country. Some tools are non-standardized; healthy lifestyle was assessed unsystematically; we did not adjust demographic variables; and in residents, we did not assess their medical condition to see the exact difference in suicidal behavior due to their status of medical condition. Exclusion criteria of a previous diagnosis of depression and of not being able to understand may have underestimated the suicidal behavior and depression symptoms.

## Conclusions

The prevalence of suicidal behavior was high in the study area with almost a double prevalence among participants who sought medical care compared with residents in the community. Therefore, mental health services should be recommended within primary health care centers to prevent later suicide death in people who visited for medical care in resource-limited settings. At least clinicians who are working in primary health care settings should assess suicidal behavior in patients who visit for medical help. According to the results of this study, more attention should be given to women visiting for checkup or care, patients who visit for medical care, and especially women who report having been abused (physical/verbal). Feeling stigmatized and depressive symptoms should be identified as risk factors for suicidal behavior, whereas having a healthy lifestyle, such as regular meal of fruits and vegetables, doing physical exercises regularly, and use of public health insurance should be supported for reducing risk of suicidal behavior.

Our finding helps as primary supportive evidence for health policymakers to recommend integration of mental health services within the primary health care level in resource-limited settings; it is evidenced that the treatment gap ranges up to 85% among people with mental illness in LMICs (49).

## Data Availability Statement

The raw data supporting the conclusions of this article will be made available by the authors, without undue reservation.

## Ethics Statement

The studies involving human participants were reviewed and approved by Ethical Review Committee of the College of Medicine and Health Sciences, Bahir Dar University. The patients/participants provided their written informed consent to participate in this study.

## Author Contributions

All authors perform the design of the study, statistical analyses, and draft the manuscript.

## Conflict of Interest

The authors declare that the research was conducted in the absence of any commercial or financial relationships that could be construed as a potential conflict of interest.

## Publisher's Note

All claims expressed in this article are solely those of the authors and do not necessarily represent those of their affiliated organizations, or those of the publisher, the editors and the reviewers. Any product that may be evaluated in this article, or claim that may be made by its manufacturer, is not guaranteed or endorsed by the publisher.

## Abbreviations

AOR: adjusted odds ratio
CI: confidence interval
HADS: hospital anxiety and depression scale
LMICs: low- and middle-income countries
SBQ-R: suicide behaviors questionnaire-revised.
